# Staying in touch with the endocytic network: The importance of contacts for cholesterol transport

**DOI:** 10.1111/tra.12726

**Published:** 2020-03-31

**Authors:** Andrea Martello, Fran M. Platt, Emily R. Eden

**Affiliations:** ^1^ UCL Institute of Ophthalmology London UK; ^2^ Department of Pharmacology University of Oxford Oxford UK

**Keywords:** cholesterol homeostasis, cholesterol transport, endosome, inter‐organellar, lysosome, membrane contact sites, tethers, trafficking

## Abstract

Cholesterol homeostasis is critical for cell function and human health. Cholesterol is heterogeneously distributed among cellular membranes, with the redistribution of endocytosed dietary cholesterol playing a pivotal role in the regulation of cholesterol homeostasis. While gaps remain in our understanding of intracellular dietary cholesterol transport, a highly complex network of pathways is starting to emerge, often involving inter‐dependent vesicular and non‐vesicular transport mechanisms. The last decade has seen a surge in interest in non‐vesicular transport and inter‐organellar communication at membrane contact sites. By providing platforms for protein interactions, signalling events, lipid exchange and calcium flux, membrane contact sites (MCS) are now appreciated as controlling the fate of large amounts of lipid and play central roles in the regulation and co‐ordination of endocytic trafficking. Here, we review the role of MCS in multiple pathways for cholesterol export from the endocytic pathway and highlight the intriguing interplay between vesicular and non‐vesicular transport mechanisms and relationship with neurodegenerative disease.

## Graphical abstract

Traffic of dietary cholesterol from the endocytic pathway to the ER is an essential step in cholesterol homeostasis but the mechanism of this cholesterol transport is not completely understood. Sites of contact between the membranes of endocytic and non‐endocytic organelles are emerging as important regulators of both vesicular and non‐vesicular cholesterol transport. Here, we review current understanding of these membrane contact sites and their importance in redistributing dietary cholesterol from endocytic organelles to other cellular membranes and roles in neurodegenerative disease.

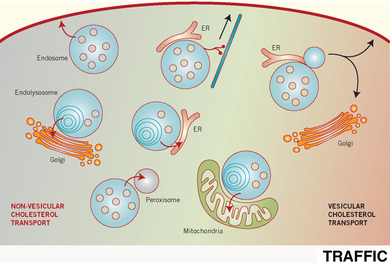

**Abbreviations**
ER: endoplasmic reticulum
LE: late endosomes
Lys: lysosomes

Cholesterol is an essential constituent of cell membranes, maintaining membrane integrity and limiting permeability, but its distribution between different cellular membranes varies considerably. Cholesterol is particularly enriched at the plasma membrane (estimated to contain approximately 60% of total cellular cholesterol^1^), whereas the endoplasmic reticulum (ER), which is the site of cholesterol sensing biosynthesis and storage, has a relatively low cholesterol content (approximately 5% of total ER lipids).^3^

Two sources of cholesterol are available to the cell—endocytosed dietary cholesterol or cholesterol synthesized de novo in the ER. While the contribution to total cholesterol from de novo synthesis is believed to outweigh that from the diet, the delivery of dietary cholesterol to the ER is important in the regulation of both sources. Dietary cholesterol is packaged into lipoprotein particles and transported in plasma, primarily in low‐density lipoprotein (LDL). LDL is comprised of a cholesterol ester/triglyceride core surrounded by a phospholipid and unesterified (“free”) cholesterol shell, with a protein component (apoB‐100) that acts as a specific ligand for the LDL receptor (LDLR). LDL enters the endocytic pathway through clathrin‐mediated LDLR endocytosis. Progressive hydrolysis by acid lipases releases free cholesterol that is delivered by the small luminal protein Niemann Pick type‐C protein 2 (NPC2) to the large transmembrane protein NPC1 on the endo/lysosomal limiting membrane.^4^ Loss of NPC1 or NPC2 activity results in an accumulation of free cholesterol in endocytic organelles and a corresponding reduction in re‐esterification by the ER‐resident enzyme ACAT, indicating impaired endosome to ER cholesterol transport. Allthough present throughout the endocytic pathway, acid lipases are more active in the increasingly acidic environment of late endosomes (LE) and lysosomes (Lys).^5^ Taken together with the localization of NPC proteins in LE and Lys, this suggests that late endocytic organelles represent a major site of cholesterol egress.

Cholesterol sensing in the ER by Insig retains SCAP‐SREBP complexes in the ER. When cholesterol transport to the ER is low, however, SCAP‐SREBP is transported to the Golgi. Proteolytic cleavage by Golgi‐resident proteases releases a soluble SREBP fragment that relocalizes to the nucleus to upregulate transcription of cholesterol biosynthesis and uptake genes.^6^ Thus, delivery of dietary cholesterol to the ER is a key step in cholesterol homeostasis, necessary for the feedback loop that regulates de novo cholesterol biosynthesis as well as LDLR gene expression and therefore uptake of dietary cholesterol. Given its importance, it is not surprising that the cell has evolved intricate mechanisms and multiple pathways for the traffic of LDL‐derived cholesterol from the endocytic pathway to the ER. Defective cholesterol transport from the endocytic pathway to the ER is associated with the lysosomal storage disorder Niemann Pick disease type‐C (NPC), where loss of function mutations in NPC1 or NPC2 prevent the egress of dietary cholesterol to the ER. This manifests as a devastating progressive neurodegenerative disease often presenting in early childhood. At a cellular level it is characterized by a marked accumulation of cholesterol and multiple sphingolipids in the late endocytic system.^7^

Delivery of dietary cholesterol to target cellular membranes involves a complex relationship between both vesicular and non‐vesicular transport mechanisms. The role of vesicular traffic in delivering material from one organelle to another has been widely studied, but only recently have we begun to appreciate the importance of non‐vesicular transport in inter‐organellar communication. Non‐vesicular lipid transport is mediated by membrane contact sites (MCS), regions of close membrane apposition (5‐30 nm) between neighbouring organelles. MCS are stabilized by tethering complexes that maintain close proximity between the opposing membranes, but without membrane fusion. These tethers can often be discerned by electron microscopy, as multiple strands between the opposing membranes of the two organelles.^8^

Since their identification only a decade ago,^9^, ^10^ there has been an explosion of interest in the field of ER‐endosome/lysosome MCS, leading to major advances in our understanding of their regulation and function.^11^, ^12^, ^13^, ^14^, ^15^ The endocytic pathway introduces large amounts of proteins and lipids into the cell and we are starting to recognize the importance of regulatory events that take place at the interface of endocytic organelles and the ER. However, endocytic organelles form contact sites not just with the ER but also with a variety of functionally distinct organelles. Roles for these MCS in orchestrating coordinated and highly regulated signalling and trafficking events along the endocytic pathway are rapidly emerging. Here, we will briefly review our current understanding of the regulation and molecular architecture of these MCS and go on to discuss their role in the redistribution of dietary cholesterol from the endocytic pathway to other cellular membranes and the implications of these MCS in neurodegenerative disease.

## 1 REGULATION AND COMPOSITION OF ENDOCYTIC ORGANELLE MCS

### 1.1 Endocytic organelle‐ER MCS

**Figure 1:**
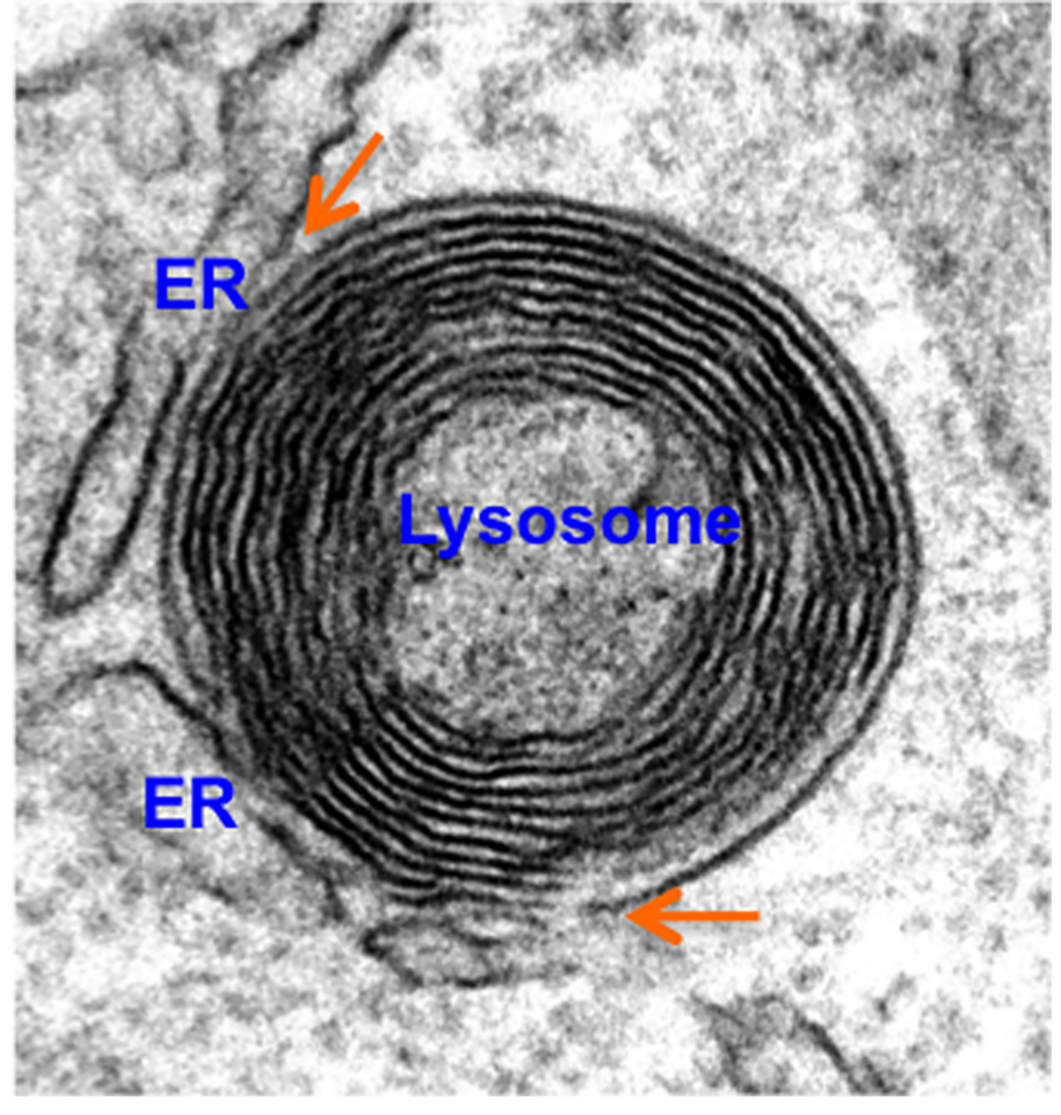
Endoplasmic reticulum (ER)‐lysosome membrane contact sites (MCS) (orange arrows). HeLa cells prepared for electron microscopy as previously described^2^ were imaged on a JOEL 1400+ TEM

The most widely studied MCS population formed by endocytic organelles is the ER‐endosome contact. The ER forms extensive MCS with endocytic organelles, which increase during endosome maturation, with approximately 90% of lysosomes estimated to form an ER contact site at any given time (eg, Figure 1).^16^, ^17^ ER‐endosome MCS provide sites for several inter‐organellar interactions, detailed in Table 1, that stabilize the MCS and are important in their regulation. Although tethers generally serve additional roles at the contact, this may not always be the case. For example, no other function at MCS has as yet been identified for the endosomal tethering protein Annexin A1. Together with its calcium‐dependent binding partner S100A11, Annexin A1 tethers MCS between the ER and a subset of endosomes that contain epidermal growth factor receptor (EGFR), providing sites for the ER‐resident phosphatase, PTP1B, to mediate effects at the endosome.^18^ PTP1B dephosphorylates both endocytosed EGFR and components of the endosomal sorting complex required for transport (ESCRT) machinery to regulate endosome maturation and receptor tyrosine kinase signalling.^9^

**Table 1:** Interactions at endoplasmic reticulum (ER)‐endocytic organelle membrane contact sites (MCS)

Endocytic organelle protein	ER binding partner	Reference
EGFR, growth factor receptor, sequestered onto endosome luminal vesicles by ESCRT‐0	*PTP1B*, tyrosine phosphatase, dephosphorylates EGFR and ESCRT‐0	9
Annexin‐A1, Ca^2+^‐dependent phospholipid binding protein and anti‐inflammatory mediator	*S100A11*, calcium‐binding protein associated with cell survival	18
IST1, ESCRT‐III component required for membrane remodelling	*M1 Spastin*, microtubule severing enzyme	19
OSBP, cholesterol and PI4P lipid transport protein; SNX2, part of retromer VPS13C, lipid transport protein at LE/Lys and lipid droplets	*VAP*, on cytoplasmic face of ER, binds proteins on a variety of organelles, including FFAT motif‐containing proteins, to tether multiple MCS populations	20, 21
Rab7, small GTPase, marks LE/Lys PI3P, endocytic membrane phospholipid	*Protrudin*, binds VAP, recruits Kinesin‐1 to LE/Lys by coincident binding of Rab7/PI3P	22
Rab7, LE/Lys biogenesis, traffic, positioning and function regulator	*PDZD8*, lipid transport protein also at ER‐mitochondria MCS	23
Coronin 1C, Actin‐regulating protein	*TMCC*, on peripheral ER, implicated in ER organization	13
ORP1L, oxysterol binding protein family, interacts with Rab7 on LE/Lys and transports cholesterol at MCS. Role in endosomal positioning through RAB7/RILP complex	*VAP/MOSBP2*, MSP domain‐containing proteins, bind FFAT motif‐containing proteins, including ORP1L on other organelles to tether MCS	10, 24
STARD3 (MLN64)/ STARD3NL (MENTHO), LE/Lys proteins that can dimerise through sterol‐binding transmembrane MENTAL domain. STARD3 mediates ER to LE/Lys cholesterol transport and mitochondrial cholesterol import	*VAP/MOSBP2*, as above, interact with STARD3 and STARD3NL through their FFAT motifs	24, 25
NPC1, LE/Lys membrane protein required for cholesterol egress	*Gramd1b/ORP5*, sterol and phospholipid transport proteins	2, 26

The MCS that endocytic organelles form with the ER are heavily influenced by their lipid environment. One potential mechanism for this sterol sensitivity is the conformational change that the late endosomal sterol‐binding protein ORP1L is reported to undergo when its oxysterol binding domain is in a sterol‐bound state. ORP1L contains a FFAT motif (two phenylalanines in an acidic tract), through which it can interact with the MSP domain of ER‐localized tethering proteins VAP^10^ (VAMP‐associated protein) and MOSPD2.^24^ Under conditions of low cholesterol in the endocytic pathway, ORP1L's FFAT motif is exposed for binding VAP (or MOSPD2) on the ER, with an associated increase in ER‐endosome connections when cells are cultured with delipidated serum.^10^ Another late endosomal sterol binding, FFAT motif‐containing protein, STARD3, is also implicated in tethering ER‐endosome contacts. However, whereas VAP or MOSPD2 silencing reduces ER‐endosome MCS, depletion of STARD3 or ORP1L has little effect on MCS formation, suggesting some redundancy between these and other VAP‐binding endosomal tethering proteins, including OSBP and VPS13C as well as possibly others that are yet to be identified. In addition to stabilizing the MCS, STARD3 and ORP1L can mediate the transport of newly synthesized cholesterol from the ER to endosomes to support endosome maturation.^18^, ^27^ Interestingly, both proteins have also been implicated, under certain circumstances, in traffic of LDL‐derived cholesterol from LE/Lys to ER (discussed in more detail below), suggesting functionality for ER‐endocytic organelle contact sites in bidirectional transport of sterols.

Unlike STARD3, which is anchored to endosomal membranes by its MENTAL domain, ORP1L is recruited to LE by the small GTPase, Rab7, which plays a number of key roles in late endocytic membrane traffic. The extended contact between Rab7‐positive LE/Lys compared with earlier (Rab7‐negative) endosomes^16^ suggests a central role for Rab7 in orchestrating these connections and two separate studies have described roles for endosomal Rab7 in tethering MCS through interaction with ER membrane proteins. First, Raiborg et al demonstrated Rab7 interaction with the ER protein protrudin^22^ and more recently Rab7 was found to interact with PDZD8 on the ER.^23^ Rab7 cycles between an active (effector‐binding), GTP‐bound state on LE/Lys membranes and an inactive, GDP‐bound state, which, through interaction with GDI, is retrieved from the membrane and held in the cytosol. The significance of Rab7 nucleotide binding in MCS formation is becoming increasingly apparent. Although Rab7 was found to bind ORP1L independently of GTP/GDP‐binding state, both protrudin and PDZD8 specifically bind GTP‐bound Rab7 and NPC1 was recently also shown to interact with GTP‐bound Rab7.^28^ Moreover, a recent study demonstrated reduced ER‐LE/Lys contact when GTP‐bound Rab7 was decreased, whereas increasing Rab7‐GTP, by inhibition of the Rab7 GAP TBC1D15, expanded the MCS.^29^

### 1.2 MCS with other organelles

Endocytic organelles have been shown to make contact with most organelles in the cell, including the Golgi, peroxisomes and mitochondria. While much less is known about the architecture of these MCS, progress has been made and several proteins with roles in contact site formation or function (or both) have been identified. Of these, the MCS between endocytic organelles and Golgi are among the better defined. Endosome/lysosome‐Golgi MCS were first identified in response to nutrient sensing. Under conditions of starvation, a tumour suppressor protein, folliculin, associates with lysosomes to drive lysosome MCS with Rab34‐positive Golgi membranes through direct interaction with the Rab34 effector RILP, with a consequent retention of lysosomes in the peri‐nuclear compartment.^30^ Similarly, nutrient‐sensing regulates mTORC localization to lysosomes, where its activity is regulated by a Golgi‐localised small GTPase, Rheb at lysosome‐Golgi MCS.^31^

Mitochondria crosstalk with the endocytic pathway, especially through the activity of AMPK, is central to the coordination of mitochondrial and lysosomal function^32^ and it has been suggested that disruption of lysosome‐mitochondria signalling may contribute to Alzheimer's disease.^33^ In addition, a number of functions have been described for physical connections between mitochondria and the endocytic pathway. Mitochondria‐endosome MCS can facilitate iron transfer in erythroid cells. As a means of avoiding cytosolic oxidation, developing erythroid cells can deliver endocytosed iron to mitochondria for haeme biosynthesis at dynamic MCS between the two organelles.^34^ These MCS were also identified in epithelial cells, where endosomal iron content regulates the dynamics of the MCS.^35^ Mitochondria‐lysosome contacts have been described in cells under conditions of hypoxia, likely functioning in lysosome‐mediated degradation of damaged protein on the outer mitochondria membrane.^36^ Interestingly, as well as playing a central role in the formation of ER‐LE/Lys contacts, Rab proteins have also been implicated in mitochondrial connections with the endocytic pathway. Hsu et al described reversible translocation of Rab5 from early endosomes to mitochondria at MCS under conditions of oxidative stress^37^ and active membrane‐bound Rab7 was found to promote mitochondria‐lysosome MCS that mark sites of mitochondrial fission.^38^ Moreover, the role of Vps13A, which interacts with Rab7 on LE/Lys, in lysosomal degradation was recently linked to its localization at the mitochondria‐LE/Lys interface.^39^

## 2 ROLE OF MCS IN CELLULAR DISTRIBUTION OF LDL‐DERIVED CHOLESTEROL

### 2.1 Endosome to plasma membrane cholesterol transport

The majority of LDL‐derived cholesterol is reported to be trafficked to the PM, to safeguard supply, before onward transport of excess cholesterol to the ER for esterification and storage in lipid droplets.^40^ No global mechanism for lysosome to plasma membrane cholesterol transport has been identified and it is likely that multiple environment/cell type‐dependent pathways may operate in parallel. One of the better defined of these is the Rab8‐dependent vesicular transport of LDL‐derived cholesterol along cortical actin to focal adhesion sites at the plasma membrane.^41^ Rab8 is recruited to LE/Lys by NPC1 and mediates transport of CD63‐positive vesicles enriched in cholesterol for Myo5b‐dependent docking at the plasma membrane. Rab8‐dependent cholesterol delivery to the plasma membrane was found to be important for cell migration, but interestingly, these LE/Lys‐derived vesicles did not fuse with the plasma membrane, suggesting non‐vesicular transport across as yet uncharacterized endosome‐plasma membrane MCS. Although not thought to operate at MCS, the oxysterol binding protein (OSBP)‐related protein 2 (ORP2) has been proposed to extract cholesterol from endocytic organelles for delivery to the plasma membrane^42^ in exchange for PI(4,5)P_2,_ 43 possibly in a mechanism analogous to that mediated by OSBP at the Golgi‐ER interface.^44^

Another potential pathway for delivery of LDL‐derived cholesterol to the plasma membrane culminates in endosome‐plasma membrane fusion. ER‐localized protrudin tethers ER‐endosome MCS through coincident binding of Rab7 and PI_3_P on LE. At these contacts, the microtubule motor protein kinesin‐1 is transferred from protrudin to the late endosomal motor adaptor FYCO1 to mediate plus end directed transport of LE to the plasma membrane. In contrast to the Rab8‐dependent pathway, however, these endosomes undergo synaptotagminVII‐dependent fusion with the plasma membrane to support membrane protrusion and neurite outgrowth. Although the impact of this pathway on cholesterol distribution has not yet been explored, it likely offers a mechanism of sterol delivery from the limiting membrane of LE to the plasma membrane.

The endocytic recycling compartment is estimated to contain approximately 30% of total cellular cholesterol, the majority of which is delivered to the plasma membrane in an ATP‐independent manner, indicative of non‐vesicular transport.^45^ It was more recently shown that the soluble sterol transport protein, STARD4, is responsible for mediating delivery of cholesterol from recycling endosomes to the plasma membrane for onward transport to the ER.^46^ Recycling endosomes are sorted away from the degradative (lysosomal) pathway in a complex process involving endosome tubulation and fission. Several studies have implicated ER‐endosome MCS in the fission of budding endosomes,^13^, ^19^, ^20^ mostly with respect to retrograde transport to the Golgi (discussed below), but the same mechanisms are believed to define fission events for recycling to the plasma membrane. Thus, although not yet directly demonstrated, it is likely that MCS‐driven fission of endosomal recycling tubules contributes to LDL‐derived cholesterol transport to the plasma membrane, with STARD4 and likely other sterol transporters mediating non‐vesicular sterol transport from recycling endosome to the plasma membrane at MCS.

### 2.2 Endosome to Golgi sterol transport

Several lines of evidence point to the Golgi as an intermediate on the endosome to ER cholesterol transport pathway. Depletion of SNARE proteins involved in vesicular traffic between the endosome and the TGN^47^ inhibited cholesterol transport from the lysosome to the TGN and resulted in the partial inhibition of cholesterol re‐esterification in the ER.^48^ Moreover, interfering with endosome‐Golgi retrograde vesicular transport by depletion of Golgi‐associated retrograde protein (GARP) resulted in cholesterol accumulation in the LEs/Lys.^49^ Together, these findings demonstrate a significant contribution to cholesterol efflux from the endocytic pathway by retrograde transport to the Golgi. There is now growing evidence that ER‐endosome MCS are important in the regulation of vesicular transport from endosomes to the Golgi.

The finding in 2014, that MCS define the position and timing of endosomal fission,^50^ has since been confirmed by a number of studies identifying a role for ER‐endosome contacts in the fission of tubular endosomes for retrograde transport to the TGN. This process is dependent on the WASH complex that regulates actin nucleation on endosomes. A recent study identified an ER transmembrane protein, TMCC1, as being in close proximity to the WASH subunit FAM21.^13^ The authors went on to show that TMCC1 is recruited to budding endosomes by the actin‐binding protein Coronin1C. TMCC1‐GFP was enriched on ER tubules at Rab7‐positive budding endosomes and endosomal fission was compromised on TMCC1 depletion, with an associated defect in retrograde transport. Moreover, depletion of Coronin1C resulted in a loss of both ER‐endosome MCS and endosomal fission. Another FAM21‐proximal protein on recycling endosomes, SNX2 is also involved in regulating MCS through interaction with VAP on the ER to mediate endosomal budding, for transport to the TGN.^20^ VAP‐SNX2 interaction tethers MCS that provide platforms for endosomal OSBP to transfer PI4P to the ER for dephosphorylation by Sac1.^20^ That endosomal fission was dependent on OSBP, which transfers PI4P at the contact, suggests that a lipid imbalance can inhibit the fission process. Another possible mechanism for the role of MCS in endosomal fission involves the ESCRTIII component IST1, which was found to interact with ER‐localized microtubule severing enzyme, M1‐spastin, to mediate endosomal fission. Mutant M1‐spastin prevented IST1 binding, with the consequent reduction in MCS, endosome fission and retrograde transport having a downstream effect on lysosomal dysfunction. Spastin mutations can cause hereditary spastic paraplegia (HSP); thus, defects in MCS‐mediated endosomal fission and downstream effects on lysosomal function were proposed to underlie axonal degeneration in HSP. ESCRTIII proteins have known roles in membrane constriction and fission, but it has also been proposed that the recruitment of ER to budding/tubular endosomes at MCS might itself serve to constrict the endosomal membrane with sufficient mechanical force to mediate fission.^51^ Thus, since MCS define sites of endosomal fission that is required for retrograde traffic, which in turn can mediate cholesterol transport, while not yet directly demonstrated, a likely role for ER‐endosome MCS in cholesterol transport to the ER via the Golgi can be inferred.

In addition to vesicular retrograde traffic, a non‐vesicular transport mechanism from Rab11‐positive recycling endosomes to the TGN at endosome‐Golgi contacts has recently been reported. Work from Sobajima et al identified a Rab11‐GTP and OSBP binding protein, RELCH that tethers recycling endosomes to the TGN to mediate cholesterol transport.^52^ The authors demonstrated RELCH‐dependent sterol transport from recycling endosome‐like liposomes to Golgi‐like liposomes. In the absence of the Rab11‐RELCH‐OSBP complex, cholesterol accumulates in LEs/Lys. This defect in cholesterol efflux could be rescued by expression of wild‐type RELCH but not mutants unable to bind Rab11 or OSBP. The authors proposed that in addition to vesicular transport, a model of non‐vesicular cholesterol transport can operate, whereby LDL‐derived cholesterol traffics across RELCH‐dependent MCS between recycling endosomes and the TGN. This non‐vesicular transport mechanism likely functions cooperatively with the vesicular transport pathways described to mediate cholesterol efflux from the endocytic pathway to the TGN.

### 2.3 LE/Lys cholesterol transport to mitochondria

Mitochondria have the lowest abundance of sterols among cellular organelles, approximately four and 40 times less than ER and plasma membrane, respectively.^53^ But like other membranes, cholesterol is required for maintaining mitochondrial membrane integrity, regulating fluidity, permeability and membrane protein interactions. Mitochondria in specialized steroidogenic cells, have acquired the ability to use cholesterol for the synthesis of steroids, oxysterols and hepatic bile acids. In response to hormonal signals, steroidogenic cells convert cholesterol to steroids. The rate limiting step in steroids synthesis is cholesterol import in the inner mitochondrial membrane, where it is converted by the P450 side chain cleavage enzyme CYP11A1 to pregnenolone which is the steroid precursor.

In yeast, mitochondria form MCS with the vacuole (yeast lysosome), called vacuole and mitochondria patches (vCLAMPs), which are regulated by a complex consisting of the Rab7 GTPase‐like Ypt7 and one of its effectors Vps39/Vam6 subunits of the HOPS (homotypic fusion and vacuole protein sorting) tethering complex and the mitochondrial protein Tom40.^54^, ^55^, ^56^ Interestingly Tom70 and Tom5 which, together with Tom40, are part of the multi‐protein mitochondrial translocation complex, have also been implicated in inter‐organellar contact. Tom70 interacts with the ER‐bound sterol transporter Lam6/Ltc1,^57^ whereas Tom5 is involved in tethering the ER through the ER Membrane protein Complex.^58^ vCLAMPs and mitochondria‐ER contact sites, termed ER‐mitochondria encounter structure (ERMES), are reciprocally regulated; while mitochondria‐vacuole MCS extend during fermentation under high glycolysis conditions and decrease on respiratory growth with active oxidative phosphorylation, mitochondria‐ER contacts increase under the same condition.^56^ Unexpectedly, the disruption of the metabolite flux from the ER to mitochondria caused by ERMES deficiency can be bypassed by a functionally redundant pathway involving vesicular transport to the vacuole and the formation of vacuole‐mitochondria MCS by Vps13 and its mitochondria binding partner Mcp1.^55^

In mammalian cells, early evidence for a complex able to transfer cholesterol to mitochondria came from studies on STARD3. STARD3 is anchored to the cytosolic side of late endosome through an MLN64 N‐terminal (MENTAL) membrane domain highly homologous to another transmembrane protein, STARD3NL. STARD3 also possesses a START sterol binding domain at the C‐terminal. Overexpression of STARD3 in steroidogenic competent cells modestly increases pregnenolone formation and expressing STARD3‐START domain alone strongly increases steroid formation.^59^, ^60^ Expression of a fusion protein (F2‐protein) of CYP11A, ferredoxin reductase and ferredoxin‐1 with the CYP11A1 mitochondrial targeting sequence enables measurement of pregnenolone production to be used as a reflection of cholesterol import in non‐steroidogenic cells. In CHO cells expressing the CYP11A1 F2 protein, pregnenolone synthesis was decreased by 40% after STARD3 silencing, reinforcing the idea that STARD3 is involved in one of multiple pathways driving sterols to mitochondria.^61^ Progress in understanding an alternative sterol pathway to mitochondria came from seminal studies on NPC. NPC is caused by mutations in either *NPC1* or *NPC2* genes. NPC2 protein mobilizes LDL‐derived cholesterol in the lysosomal lumen and subsequently transfers it to NPC1 for egress from the limiting membrane. Depletion of NPC2 in CHO cells expressing the CYP11A1 F2‐protein, reduced pregnenolone formation to the same extent as STARD3 depletion, but combined STARD3 and NPC2 knockdown had no additional effect on pregnenolone synthesis.^62^ Conversely, mitochondria in NPC1 mutant cells have elevated cholesterol and STARD3 silencing can revert this phenotype.^61^ Ultrastructural evidence of mitochondria‐lysosome MCS in NPC1‐deficient or inhibited cells demonstrated extensive contact that can be abrogated by silencing STARD3. Interestingly depleting NCP1 or its tether partner, the ER‐localized sterol transport protein Gramd1b, is sufficient to increase the MCS,^2^ suggesting a level of regulation by lysosomal sterol content. Thus, as was found in yeast for mitochondria ‐ER vs ‐vacuole contacts, there appears to be reciprocal regulation between ER‐lysosome and mitochondria‐lysosome MCS populations, likely governed by the sterol accumulation in lysosomes on loss of ER contact.

### 2.4 LE/Lys cholesterol transport to peroxisomes

Like lysosomes, peroxisomes are organelles bound by a single membrane that participate in metabolic pathways. Under conditions of high LDL‐cholesterol in the endocytic pathway, lysosomal Synaptotagmin VII binds PI_4,5_P_2_ on the peroxisome membrane to tether connections between the two organelles.^63^ These MCS were reported to make a significant contribution to cholesterol export from the lysosome, with lysosomal cholesterol accumulation resulting on their disruption. Moreover, cells from patients with the peroxisome biogenesis disorders Infantile Refsum disease or Zellweger syndrome also accumulate cholesterol, suggesting an important role for peroxisomes in receiving LDL‐derived cholesterol.

### 2.5 Direct transport of LDL‐derived cholesterol from LE/Lys to the ER

While the majority of cholesterol transport between the endocytic pathway and the ER is believed to occur via the plasma membrane, it is estimated that direct transport mechanisms between LE/Lys and the ER account for approximately 30% of LDL‐derived cholesterol delivery to the ER.^64^, ^65^ Given the extensive contact between these organelles, coupled with the demonstration that LE/Lys sterol‐binding proteins can interact with ER proteins to extend the contact, MCS were widely predicted to serve as conduits for cholesterol transport from LE/Lys to the ER.^8^, ^66^, ^67^, ^68^, ^69^ The first real evidence for cholesterol transport at ER‐LE/Lys MCS came from a study demonstrating a role for ORP1L, previously identified as a regulator of microtubule‐dependent movement of LE/Lys,^10^, ^70^ in the transport of cholesterol from the limiting membrane of endocytic organelles to the ER.^70^ In order to fulfil this role, ORP1L must be able to bind not only sterol, but also VAP on the ER, indicating that ORP1L‐mediated cholesterol transport to the ER occurs at MCS between the two organelles.^70^ Interestingly, in addition to sterol and VAP binding, phosphatidylinositol phosphate (PIP) binding was also necessary for ORP1L's role in the transport of LDL‐derived cholesterol to the ER. This potential role for PIPs in regulating ORP1L's sterol transfer activity was confirmed by a recent study revealing allosteric regulation of ORP1L‐mediated cholesterol transport by PIPs.^71^ This study found that while ORP1L cannot transfer PIPs, PI(3,4)P_2_ and PI(4,5)P_2_ increase the rate at which ORP1L extracts cholesterol from the membrane to regulate egress of cholesterol from LE/Lys. This could underlie the inhibition of mTORC1 by PI(3,4)P_2_ accumulation on LE/Lys. LE/Lys cholesterol can activate mTORC through SCL38A9,^72^ thus, PI(3,4)P_2_ enhancement of ORL1L‐mediated cholesterol egress could result in mTORC1 inhibition.

NPC1 has an established role in cholesterol egress from late endocytic organelles and although the handover of cholesterol from NPC2 on the lumen of LE/Lys to NPC1 on the membrane, has been structurally defined,^73^ our understanding of the mechanism of NPC1‐mediated cholesterol transport remains incomplete. The recent structure of yeast NPC1 (Ncr1) sheds some light on how NPC1 may function. The lysosomal membrane is largely protected from luminal degradative enzymes by a polysaccharide matrix called the glycocalyx. The NCR1 structure revealed that luminal cholesterol is delivered from NPC2 to an NPC1 tunnel to bypass the glycocalyx for delivery to the limiting membrane.^74^ This explains how NPC1 transports cholesterol from the LE/Lys lumen to the cytosolic leaflet of the limiting membrane, but the next step in cholesterol egress, transport from the limiting membrane to the ER, has remained elusive. Two independent studies, one from our lab and the other from Meneses‐Salas et al, have gone some way to resolving this fundamental gap in our knowledge, uncovering an additional role for NPC1 in MCS formation.^2^, ^29^ Both studies found that ER‐LE/Lys MCS were reduced on loss of functional NPC1, suggesting a regulatory role for NPC1 in MCS formation. In addition to the reduced MCS on NPC1 depletion, NPC1 overexpression increased MCS. Moreover, NPC1 was enriched at the contact, where it was found to interact with an ER‐localized sterol‐transfer protein, Gramd1b,^75^ previously shown to mediate non‐vesicular transport of HDL‐derived cholesterol from the plasma membrane to the ER.^76^ Interestingly, Gramd1 proteins appear to translocate to high cholesterol content membranes, sensing cholesterol‐loading at the plasma membrane to facilitate plasma membrane to ER sterol transport,^76^, ^77^ and preferentially interacting with NPC1 under conditions of high cholesterol in the endocytic pathway to mediate lysosome to ER transport.^75^ Thus, NPC1 meets all the criteria for a tether^78^ and appears to be an important regulator of MCS formation. Reduced MCS on loss of NPC1 activity was also found to be associated with reduced GTP‐bound (active) Rab7 due to recruitment of the Rab7 GAP TBC1D15 by endosomal AnnexinA6.^29^ GTP‐Rab7 was restored on depletion of AnnexinA6, resulting in increased ER‐LE/Lys MCS and, excitingly, reversal of the cholesterol accumulation. Similarly, a Rab7 GEF that mediates GDP to GTP exchange, was recently shown to promote lysosomal cholesterol egress and delivery to the ER.^28^ Moreover, ORP1L overexpression, which also expands MCS,^10^, ^18^ also resulted in restoration of cholesterol transport from LE/Lys to the ER in NPC1‐deficient cells.^2^ Intriguingly, ORP1L VAP binding but not sterol binding capabilities were necessary to rescue the cholesterol accumulation in NPC1‐deficient cells. Thus, artificially tethering the contact is sufficient to enable cholesterol transport even in the absence of NPC1. While cholesterol may be able to shuttle across the MCS along the concentration gradient, moving from high concentrations in the LE/Lys to low concentrations in the ER, restoration of transport on AnnexinA6 silencing was dependent on STARD3, suggesting that STARD3, or an alternative lipid transfer protein, mediates sterol transport in the absence of functional NPC1. In contrast to the studies described above, an increase in MCS in mutant NPC1 fibroblasts was recently reported to mediate OSBP‐dependent sterol transport from the ER to the lysosome to activate mTORC.^79^ Two other lipid transfer proteins have also been implicated in ER‐LE/Lys MCS, both of which may contribute to cholesterol egress. The ER‐localized protein PDZD8 mediates tethering to LE/Lys through interaction with Rab7‐GTP^23^ and Vps13C, a FFAT motif containing protein, tethers LE/Lys to the ER through interaction with VAP on the ER.^21^

The role for NPC1 in MCS regulation coupled with the finding that MCS expansion can restore cholesterol transport in NPC1‐deficient cells, underscores the importance of MCS in cholesterol egress and further points to the existence of an alternative, NPC1‐independent mechanism, for cholesterol passage across the glycocalyx. NPC2 has been proposed to mediate cholesterol transfer directly to the limiting membrane, as well as to other transmembrane proteins.^80^ Moreover, NPC2‐dependent lysosome to mitochondria cholesterol transport was shown to be independent of NPC1.^62^ As the most abundant and extensively glycosylated lysosomal membrane proteins,^81^ LAMPs are the major components of the glycocalyx. Interestingly, LAMPs were found to interact with NPC proteins and can facilitate cholesterol egress through direct sterol binding.^82^ Another study found changes in the glycocalyx in NPC1‐null cells, including reduced lysosomal LAMP1 and increased lysosomal leakage of fluorescent dextran.^83^ These findings raise the possibility that in the absence of NPC1, reduced LAMP at the lysosome could result in a glycocalyx that offers less of a barrier to cholesterol reaching the limiting membrane. Alternatively, perhaps LAMP itself could compensate for NPC1 in shuttling cholesterol to the membrane. Another pathway has also been recently shown to run in parallel to NPC1‐dependent cholesterol export. The lysosomal integral membrane protein, LIMP‐2 was found to mediate a slower pathway of cholesterol transport to ER alongside NPC1‐dependent pathways.^84^ Since its luminal domain extends beyond the glycocalyx, LIMP‐2 can offer an alternative mechanism for cholesterol delivery to the lysosomal membrane.

As a key role of MCS in cholesterol egress continues to emerge, so too does an association between defects in MCS proteins and neurodegenerative disease (Table 2). The mechanistic association between MCS/lipid transport defects and neurodegenerative disease is not well understood. The post‐mitotic nature of neuronal cells is a likely contributing factor as is their dependency on the orchestrated movement of organelles up and down axons, often over considerable distances. Unlike dividing cells, neuronal cells lack the potential to dilute accumulating material afforded by cell division. Another consideration is calcium signalling, since neurons are extremely sensitive to calcium concentrations. ER‐Lysosome MCS have been heavily implicated in refilling of lysosome calcium stores^11^, ^90^ and the generation of localized calcium signals,^17^, ^91^ that may regulate the contact extent,^92^ suggesting a potential calcium‐regulated calcium signalling mechanism at MCS. Disruption of calcium signals as a consequence of MCS defects may therefore potentiate neurodegeneration. Indeed, calcium dysregulation is strongly associated with neurodegenerative diseases and moreover, acidic organelle calcium stores are depleted in NPC,^93^ likely contributing to disease pathogenesis. Sphingosine accumulation in NPC was shown to trigger calcium release from acidic organelles, possibly underlying the depleted acidic organelle calcium stores in NPC1‐deficient cells. The role of MCS in sphingolipid metabolism has not yet been explored, but sphingolipids accumulate in NPC, where MCS are reduced. Since sphingolipids are not stored in lipid droplets, sphingolipid homeostasis must be tightly regulated and sphingolipids are particularly enriched in the nervous system.

**Table 2:** Membrane contact sites proteins implicated in neurodegenerative disease

Protein	Localization/contacting organelle	Disease association
M1 Spastin	ER/endosome^19^	Hereditary spastic paraplegia
Protrudin	ER/endosome^22^	Hereditary spastic paraplegia^85^
VAP	ER/endosome^18^, ^20^, ^25^	Amyotrophic lateral sclerosis^86^
SNX2	Endosome/ER^20^	Defects in retromer implicated in Alzheimer's disease^87^
Rab7	LE&Lys/ER^22^, ^23^, ^29^ LE&Lys/mitochondria	Charcot–Marie‐Tooth 2B^88^
VPS13C	Endosome/ER^21^	Autosomal recessive Parkinsonism^89^
NPC1	LE&Lys/ER^2^	NPC^86^

In summary, we are beginning to appreciate the important role played by MCS in cholesterol homeostasis and the complex network of organelle interactions involved. By studying rare neurodegenerative diseases associated with defects in MCS formation, much is being learnt about inter‐organelle sterol trafficking. These advances are paving the way for innovative new treatment strategies.

## CONFLICT OF INTEREST

The authors confirm they have no conflicts of interest to report.
